# Molecular detection and phylogenetic tree of infectious laryngotracheitis virus in layers in Al-Diwaniyah province, Iraq

**DOI:** 10.14202/vetworld.2019.605-608

**Published:** 2019-04-25

**Authors:** Furkan Alaraji, Hasan Hammadi, Alaa Abdulaziz Abed, Yahia Ismail Khudhair

**Affiliations:** 1 Department of Pathology and Poultry Diseases, College of Veterinary Medicine, University of Al-Qadisiyah, Diwaniyah, Iraq; 2 Department of Internal and Preventive Medicine, College of Veterinary Medicine, University of Al-Qadisiyah, Diwaniyah, Iraq

**Keywords:** DNA sequencing, infectious laryngotracheitis virus, laryngotracheitis, laying hens

## Abstract

**Background and Aim:**

Infectious laryngotracheitis (ILT) of chickens is a substantial issue to be studied in Iraq because this disease is one of the most highly contagious respiratory diseases in the world caused by a herpesvirus. However, in Iraq, the ILT virus (ILTV) infection and disease have yet not been confirmed in layers, so farm owners do not vaccinate these layers. The current study aimed to document the detection and characterization of ILTV in layer hens from Al-Diwaniyah city, for the first time in Iraq, using molecular techniques like polymerase chain reaction (PCR) and sequencing.

**Materials and Methods:**

Four layer farms (15,000 unvaccinated layers/farm) in Al-Diwaniyah province, Iraq, suffered a severe ILT outbreak, was diagnosed and reported by clinical and PCR tests. This disease has been reported in Iraq, and more recently, it began to show outbreaks in Al-Diwaniyah city. The current work opted to investigate the ILTV using PCR and DNA sequencing techniques. The study targeted the p32 gene of ILTV using pooled tracheal swabs and organs including the trachea, lung, and kidneys which were collected from dead and clinically infected chickens.

**Results:**

The analyses revealed that four of six suspected field samples showed positive results by PCR. The DNA sequencing results showed the homology of the amplified fragments with the studied gene.

**Conclusion:**

This study confirmed the presence of ILTV in hens with respiratory signs during the outbreak.

## Introduction

Infectious laryngotracheitis (ILT) has gained accelerated attention worldwide because this disease can cause fundamental economic losses including decline egg production, low growth rates, and mortality [1].

ILT virus (ILTV) is susceptible to disinfectants, but it also can survive for weeks to months if protected by organic materials such as manure, deep litter, biofilms, or respiratory secretions [2]. Although ILTV has been controlled by vaccination, ILT still considered to be a big threat for poultry industry mainly in high-density poultry flock areas [3]. Of this disease, the severe form manifested in severe respiratory pain, bloody sputum, and elevated mortality levels. On the other hand, the mild form is manifested by less severe clinical signs such as mucoid tracheitis, sinusitis, and lower mortality level [4]. ILTV is conveyed horizontally which replicated primarily in the trachea and conjunctiva where it can show its manifestations [5]. In addition, ILTV can infect the trigeminal ganglia during the lytic phase that causes the latent phase of the viral infection that might stay lifelong with the animal [5]. Recent analytical studies have found the ILTV in the heart, liver, spleen, lung, kidney, tongue, thymus, proventriculus, pancreas, duodenum, small intestine, large intestine, cecum, cecal tonsils, bursa, and brain [6].

Studies have found that herpes simplex virus-1 gene has a 63 homology open reading frame sites out of 77 of the whole genome. ILTV detection by isolation techniques is time-consuming, while other methods such as serological tests such as fluorescent antibody technique, enzyme-linked immunosorbent assay, serum neutralization, and agar gel immunodiffusion are generally of low sensitivity and laborious. Nowadays, identification of the viral DNA by conventional polymerase chain reaction (PCR) such as using infected cell polypeptide 4 (ICP4) gene [7,8] or real-time PCR is the preferred method [9]. In acute forms of the disease, infection spreads rapidly, and mortality rates may reach up to 50% [10]. In such circumstances, a reliable and prompt diagnosis is important and doable using molecular diagnostic techniques [9,11] including PCR. For those reasons and more such as reduced risk of carryover contamination, the real-time PCR has acquired its prevalence [12].

The current study aimed to document the detection and characterization of ILTV in layer hens from Al-Diwaniyah city, for the 1^st^ time in Iraq, using molecular techniques like PCR and sequencing.

## Materials and Methods

### Ethical approval

The samples were collected from the outbreak area. The research was done according to global and national ethical criteria for animal care and use.

### Sampling and poultry farms

In this study, the isolation of ILT virus was conducted by collecting the samples pooled tracheal swabs and organs such as the tracheas, lungs, and kidneys, from four layer farms (15,000 unvaccinated layers/farm) that suffered respiratory problems and 17-30% mortality rate. The study was conducted during January 2018-May 2018 in Al-Diwaniyah province after severe outbreak. After doing the postmortem examination on infected layers, the suspected diagnosis was ILT. Six positive samples were used to perform additional analyses to confirm the ILTV.

### Extraction of DNA

DNA Extraction Kit (gSYNC, Taiwan) was used for the extraction of DNA from pooled samples of layer hens suspect of ILTV infection.

### PCR

**Figure-1:**
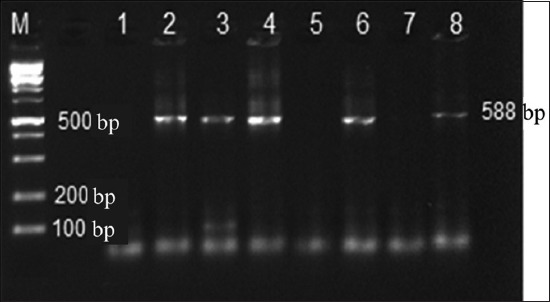
The polymerase chain reaction results of the p32 gene of infectious laryngotracheitis virus (ILTV) are shown in four samples at 588-bp fragment. M=100-bp DNA ladder; 1=Negative control; 2=Positive control (ILTV reference strain); 3-8=field strains isolated.

Primers, designed previously by Vögtlin *et al*. [13], were used to target a fragment of p32 gene of ILTV. These primers named of ILTU2 5-CTA CGTG CTGGG CTCT AATCC-3 and ILTL2 5-AAACTCT CGGG TGGCT ACTGC-3 produced a 588-bp. The amplification was done by adding 5 µL of the DNA to the PCR mix (Invitrogen™), and the total volume of the reaction was set up to 50 µL. The reaction was carried out in a Multigene Thermal Cycler (Labnet, USA). The denaturation temperature was 95°C for 5 min, then 35 cycles of 1 min at same temperature. The extension temperature was 72°C for 10 min. PCR product was analyzed by electrophoresis in 1.5% agarose gel and visualized under UV light for the 588-bp fragment as shown in Figure-1.

### DNA sequencing

To confirm the specificity of the amplicons, two samples of the PCR products were sent for DNA sequencing, using the same primers mentioned above to laboratory in Korea (Macrogen, Seoul, Korea). BLASTn in NCBI website was used to evaluate the sequence homology of ILTV with sequences from GenBank (www.ncbi.nlm.nih.gov/BLAST).

## Results

### PCR

The fragment of 588bp of the p32 gene of ILTV is shown in four samples (Figure-1). The positive band also appears in the reference strain used as a positive control, and the figure also reveals no bands detected in the negative control.

### DNA sequencing

**Figure-2:**
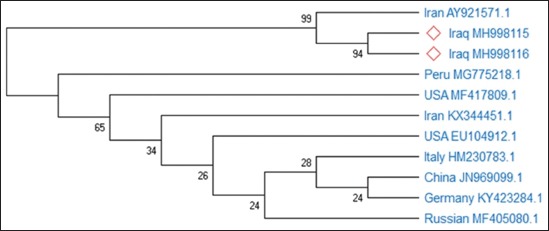
Phylogenic tree of two infectious laryngotracheitis Iraqi isolates using MEGA X program software.

Phylogenetic analysis and divergence relationship of the two selected Iraqi isolates that are illustrated in Figure-2 and Table-1 revealed that the ILT Iraqi viruses (MH998115 and MH998116) have a closer relationship with Iranian strain (AY921571) of nucleotide homology reached up to 99%.

**Table-1:** Percentage of nucleotide divergence of 12 ILT isolates calculated by MEGA X program software.

ban AY921571.1										
USA_EU104912.1	0.00									
Italy_H\I230783.1	0.00	0.00								
China_JN969099.1	0.00	0.00	0.00							
Iran_KX344451.1	0.00	0.00	0.00	0.00						
Germany_KY423284.1	0.00	0.00	0.00	0.00	0.00					
Russian_MF405080 1	0.00	0.00	0.00	0.00	0.00	0.00				
USA_MF417809.1	0.00	0.00	0.00	0.00	0.00	0.00	0.00			
Peni_MG775218 I	0.00	0.00	0.00	0.00	0.00	0.00	0.00	0.00		
MH998115_Iraq	0.00	0.01	0.01	0.02	0.01	0.01	0.01	0.01	0.01	
MH998116 Iraq	0.00	0.01	0.01	0.01	0.01	0.01	0.01	0.01	0.01	0.00

## Discussion

Avian ILT was classified as a specific viral disease of chickens in the United States in 1926 [14]. It was reported as a problem throughout the world, as it was reported in Lebanon [15] and Saudi Arabia [16] as a reason of serious economic crises in layers and broilers. In Iraq, there is controversial information about the disease, and no study has registered it before. This study is the first in the country to document an outbreak of respiratory disease with the detection of ILTV using PCR and sequencing. According to the PCR results, four samples showed positive results out of the six samples that were collected. To confirm the specificity of the PCR results, PCR products of two samples were submitted to DNA sequencing. Rapid and conventional diagnosis is helpful in case of an outbreak. Thus, PCR was a method of choice to confirm ILTV infection in chickens with clinical signs suggestive of this disease since results can be obtained in <24 h [17]. Nevertheless, additional histopathology is recommended to confirm the disease [3,8]. Besides, sequencing of the PCR came out to confirm the PCR results specifically and safely. To identify field and vaccine strains, the DNA sequencing technique can be applied depending on the availability of some molecular markers [17]. Some recent work [7,8] identified ILT infection in layers using ICP4 gene giving trustful results; however, our methodology provided reliable results as our samples were obtained from unvaccinated layers eliminating false-positive results. For histopathological diagnosis, the farm owners performed the notification of the outbreak 1 month after the onset of clinical signs, and only swabs were available to perform tests.

## Conclusion

The current work confirms the diagnosis of ILTV in layer hens from farms that experienced respiratory disease outbreak in the region of Al-Diwaniyah city, Iraq. Other extensive studies are needed to conduct epidemiological surveillance in this region to determine the incidence, prevalence, and economic impact of the disease. Furthermore, it is of importance to detect the sources of the viruses to be field or vaccine strains to gain valuable information about the origin of the disease and routes of transmission. The phylogenetic results of the studied samples of this outbreak suggested that ILTV was brought by carrier birds and spread rapidly to neighboring flocks due to the stressed situation of non-vaccinated layers. In conclusion and according to the study, it is highly recommended to improve the biosecurity measures in Iraq to avoid ILT infection.

## Authors’ Contributions

FA and HH collected the samples, recorded the case history, monitored the cases, making an experimental design, performed the PCR, and drafted the manuscript. AAA and YIK prepared the phylogenetic tree and searched the literature for citation purposes. All authors read and approved the final manuscript.
